# Comparing caries risk profiles between 5- and 10- year-old children with cleft lip and/or palate and non-cleft controls

**DOI:** 10.1186/s12903-015-0067-x

**Published:** 2015-07-25

**Authors:** Anna Lena Sundell, Christer Ullbro, Agneta Marcusson, Svante Twetman

**Affiliations:** Department of Pediatric Dentistry, Institute for Postgraduate Dental Education, Box 1030, SE 551 11 Jönköping, Sweden; Institute for Clinical Dentistry, UiT The Arctic University of Norway, Tromsø, Norway; Department of Dentofacial Orthopedics, Maxillofacial Unit, Linköping University Hospital, Linköping, Sweden; Department of Odontology, Faculty of Health and Medical Sciences, University of Copenhagen, Copenhagen, Denmark

**Keywords:** Cleft lip, Cleft palate, Cleft lip and/or palate, Caries risk, Cariogram, Children

## Abstract

**Background:**

Previous studies have suggested that children with oral clefts may have higher caries prevalence in comparison with non-cleft controls but the relative importance of the potential risk factors is not clear. The aim of this study was to compare the caries risk profiles in a group of cleft lip and/or palate (CL(P)) children with non-cleft controls in the same age using a computerized caries risk assessment model.

**Methods:**

The study group consisted of 133 children with CL(P) (77 subjects aged 5 years and 56 aged 10 years) and 297 non-cleft controls (133 aged 5 years and 164 aged 10 years). A questionnaire was used to collect data concerning the child’s oral hygiene routines, dietary habits and fluoride exposure. Oral hygiene was assessed using Quigley-Hein plaque Index and the caries prevalence and frequency was scored according to the International Caries Detection and Assessment System. Whole saliva samples were analyzed for mutans streptococci, lactobacilli, buffering capacity and secretion rate. The risk factors and risk profiles were compared between the groups with aid of Cariogram and the estimated risk for future caries was categorized as “high” or “low”.

**Results:**

Children with CL(P) (the entire study group) had significantly higher counts of salivary lactobacilli (*p* < 0.05) and displayed less good oral hygiene (*p* < 0.05). More 10-year-old children in the CL(P) group had low secretion rate but this difference was not significant. The average chance to avoid caries ranged from 59 to 67 % but there were no significant differences between the groups. The odds of being categorized with high caries risk in the CL(P) group was significantly elevated (*OR* = 1.89; 95 % *CI* = 1.25–2.86). In both groups, children in the high risk category had a higher caries experience than those with low risk.

**Conclusion:**

Children with CL(P) displayed increased odds of being categorized at high caries risk with impaired oral hygiene and elevated salivary lactobacilli counts as most influential factors. The results suggest that a caries risk assessment model should be applied in the routine CL(P) care as a basis for the clinical decision-making and implementation of primary and secondary caries prevention.

## Background

Cleft lip and/or palate (CL(P)) is the most common congenital craniofacial deformity, affecting nearly two in every 1.000 newborns in Sweden [1]. The association between CL(P) and dental caries in children is not fully clear but a number of studies indicate a higher caries prevalence in children with different oral clefts in comparison with non-cleft controls [2–6]. Several factors can contribute to this higher susceptibility such as impaired oral hygiene [2, 5, 7, 8], enamel hypoplasia [9, 10] and early colonization of caries-associated microorganisms [11]. Furthermore, parents to children with CL(P) tend to overindulge the children and offer them sucrose-containing food and snacks as a compensation for their medical condition [12, 13]. The prolonged oral clearance time in children with oral clefts may also contribute to a cariogenic environment [14]. The role and relative importance of the potential risk factors are however not clear. The Cariogram caries risk assessment software offers an algorithm-based model built on ten different caries risk factors to estimate the relative impact of common risk factors and calculate the chance to avoid caries in the near future [15]. The model has previously been validated as useful in schoolchildren [16, 17] and shows a high sensitivity for caries development in preschool children [18]. The aim of the present study was therefore to apply the Cariogram model in group of 5- and 10-year-old children with CL(P) to unveil the caries risk. The null hypothesis was that the risk would not differ from that of children in the same age without CL(P).

## Methods

The project was approved by the regional Ethics committee in Linköping (Dnr 2011/252-31 and Dnr 2012/304-32).

### Study groups

*CL(P)group* - All 5- and 10- year- old children born with any type of cleft lip/ and or palate attending two regional cleft centers in Sweden (Linköping and Gothenburg), were eligible for the study. The custodians and their children were informed about the study by mail or when visiting the cleft center. Non-responders were re-contacted per mail and/or per telephone 2–4 weeks after the first information. In total, 258 children were invited and 139 children (54 %) were clinically examined after informed consent. The reasons for the attrition were *i)* no response to the invitation (*n* = 82), *ii)* declined to consent (*n* = 35), and *iii)* no cooperation (*n* = 2). Out of the 139 examined children, one child had the diagnose Pierre Robin sequence, two had Attention-Deficit/Hyperactivity Disorder, two had autism, sixteen were asthmatic, two had heart problems, one had enteral nutrition, and one had posttraumatic stress syndrome.

*Control group* – 5- and 10- year- old children born without any type of oral cleft were randomly selected from six different public dental service clinics located in the same geographic regions and with the same sociodemographic characteristics as the children with CL(P). The dental examination was timed with the regular dental recall visit at the clinics and the parents agreed that their children participated in the study. In total, 313 non-cleft children were examined. In this group, two children had Attention-Deficit/Hyperactivity Disorder, one had autism, twenty-two had been given the diagnose asthma, five had heart problems, three had epilepsies and one had diabetes. The parents gave written consent, before the examination, for their children’s participation in the study.

**Fig. 1:**
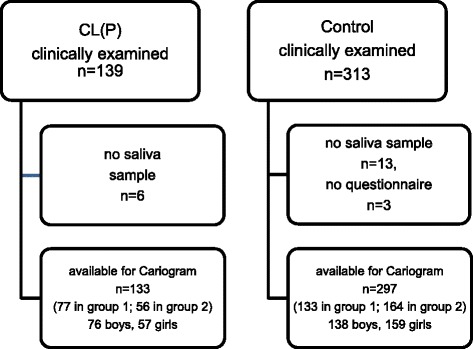
Flow-chart of inclusion and drop-out. Group1; 5-year-olds and group 2;10-year- olds

At the time of examination, 6 children in the CL(P) group and 13 children in the control group did not cooperate with the saliva collection. In addition, the parents of 3 children in the control group were unable to complete the questionnaire due to language problems. Thus, the final material for Cariogram processing consisted of 133 children with CL(P) and 297 non-cleft children in the same ages as detailed in Fig. 1.

### Clinical examination

First, the children and their custodians were asked to fill in a questionnaire developed for this study concerning oral hygiene routines, dietary habits and fluoride exposure. The clinical examinations were carried out by one of two experienced pediatric dentists in a fully equipped dental setting. The amount of plaque on the buccal and lingual surfaces of the teeth, in the first and fourth quadrants was scored after staining with erythrosine according to the modified Quigley-Hein plaque Index (QH) [19, 20]. Before the caries examination, a professional mechanical tooth cleaning with a rubber cup and prophylactic paste was done. Caries registration were made according to International Caries Detection and Assessment System (ICDAS-II) [21]. No radiographs were taken and a molar with fissure sealant was recorded as sound. Before the start of the study, the examiners were calibrated using the ICDAS-II criteria and the registrations were validated through a re-examination of 15 children within a period of one month. The intra- and inter-examiner agreement produced an index of positive consensus 0.75 (examiner 1), 0.92 (examiner 2) and 0.97, respectively. The corresponding values for a negative consensus were 0.99 (examiner 1), 1.00 (examiner 2) and 1.00.

### Saliva tests

Paraffin-stimulated whole saliva was collected in connection to the clinical examination. The parents were instructed that their child should refrain from eating, drinking and tooth brushing at least 2 h before the dental visit. The sampling was interrupted when 3.5 ml saliva was collected or when the child refused to collaborate any longer. The secretion rate was estimated in milliliter per minute. Buffer capacity (Dentobuff® Strip), mutans streptococci (Dentocult® SM-Strip mutans) and lactobacilli (Dentocult®LB) counts were estimated with commercial chair-side tests purchased from Orion Diagnostica, Espoo, Finland. All tests were handled according to the manufacturer’s instructions.

### Risk profile and risk category

The caries risk was evaluated with an algorithm-based software, the Cariogram [22]. The obtained data from the clinical examination, the questionnaires and the salivary tests of each child were entered in the computer program to calculate a graphic sector indicating the “chance to avoid caries in the future”. The “clinical judgment” was set as 1 and the standard mode was used for country/area and group. However, since the model originally was constructed for adults, the clinical scores were modified to fit the present age groups. The previous caries experience, including initial lesions, was scored 1 if caries free, score 2 for dmfs/DMFS 1–2, and score 3 in the event of ≥3 dmfs/DMFS. Concerning general diseases, medically compromised children (CL(P), asthma, heart diseases, obesity, diabetes, Attention-Deficit/Hyperactivity Disorder and autism) were scored 1 and score 2 was used when two or more related diseases were present. Plaque amount was scored 0 when QH was 0–1, score 1 for QH 1.1-2, score 2 for QH 2.1-3.5 and score 3 for QH 3.51-5. In the younger age group, the variable “salivary secretion rate” was omitted due to the difficulty to adequately determine the saliva secretion rate. In the older age group, the saliva secretion rate was scored 0 when the secretion rate was over 0.5 ml/min, score 1 for 0.49-0.25 ml/min and score 2 when under 0.25 ml/min. All other factors were handled according to the Cariogram manual. The program presents a pie diagram with five sectors in which “circumstances” are based on caries experience and related diseases, “bacteria” is based on amount of plaque and mutans streptococci, “susceptibility” on fluoride exposure, saliva secretion and saliva buffer capacity, while “diet” is based on diet contents, diet frequency and the amount of lactobacilli. The fifth sector symbolizes the “chance of avoiding caries” in the near future. In this study, only two risk categories were used; “high” = 0-60 % chance to avoid caries, and “low” = 61-100 % chance to avoid caries in the near future.

### Statistical methods

All data were processed with IBM-SPSS software (version 20, Chicago, IL, USA). Descriptive statistics were used to summarize caries risk profiles, risk categories and caries frequency. The difference in risk variables were tested using Pearson chi-square test while caries data were subjected to non-parametric Mann–Whitney test due to the skewed distribution. The difference between risk profiles were tested using Cochrane-Mantel Haenszel test. The level of statistical significance was set at 5 % (*p* < 0.05).

## Results

The distribution of the caries risk variables are shown in Table 1. Children with CL(P) (the entire study group) harbored significantly higher counts of salivary lactobacilli (*p* < 0.05) and displayed less good oral hygiene (*p* < 0.05). More children in the CL(P) group had low secretion rate but this difference was not significant. The average Cariogram sectors for children with and without CL(P) in the different age groups are presented in Table 2. The estimated average chance to avoid caries ranged from 59 to 67 % but there were no significant differences between the groups. However, as seen in Table 3, the odds for being categorized with high caries risk in the CL(P) group was significantly elevated (*OR* = 1.89; 95 % *CI* = 1.25-2.86). The caries experience in the high and low risk categories is summarized in Table 4. There was a clear tendency to increased caries scores in the high risk group but the difference was not statistically significant for the cleft children.

**Table 1:** Distribution of the Cariogram risk variables expressed as percent in both ages groups

	CL(P)			Control			*p*-value
	Low	Medium	High	Low	Medium	High	
Mutans streptococci	75	21	4	83	15	2	NS
Lactobacilli	81	17	2	91	6	3	0.001
Buffering capacity	7	29	64	11	37	52	NS
Plaque/oral hygiene	45	51	4	57	42	1	0.02
Intake frequency	-	84	16	1	79	20	NS
Fluoride exposure	-	71	29	1	74	25	NS
Saliva secretion rate	12	12	76	4	14	82	NS

Statistic method: Pearson chi-square test

Mutans streptococci counts: Low = <10^5^ CFU; Medium = 10^5^- < 10^6^ CFU; High = ≥10^6^ CFU

Lactobacilli counts: Low = ≤10^4^ CFU; Medium = 10^5^ CFU; High = ≥10^6^ CFU

Buffer capacity (final pH): Low = blue; Medium = green; High = yellow

Oral hygiene: Low = QH 0.0-2.0; Medium = QH 2.1-3.4; High = QH 3.5-5.0

Intake frequency: Low = ≤4 per day; Medium = 5-6 per day, High = ≥7 per day

Fluoride exposure: Low = no fluoride used; Medium = fluoride toothpaste is used; High = fluoride supplements are used in combination with fluoride toothpaste

Saliva secretion rate (only for 10-year old children): Low = <0.25 ml/min; Medium = 0.25-0.49 ml/min; High = > 0.5 ml/min

NS no statistically significant difference

**Table 2:** The Cariogram sectors for two age-groups of children with and without CL(P). The values in the table denote mean percent (SD)

	5-year-olds		10-year-olds	
	CL(P) *n* = 77	Control *n* = 133	CL(P) *n* = 56	Control *n* = 164
Chance to avoid caries	59 (14)	61 (12)	63 (21)	67 (17)
Diet	11 (5)	12 (5)	11 (6)	10 (6)
Bacteria	10 (6)	9 (5)	10 (7)	8 (7)
Susceptibility	13 (5)	15 (6)	10 (10)	10 (7)
Circumstances	7 (2)	4 (2)	7 (3)	5 (3)

**Table 3:** Distribution of children with increased caries risk versus low risk the two groups assessed with the Cariogram model. The values denote the number of subjects

Groups	n	Risk^a^	Low risk^b^	OR	95 % Cl
5-year-olds					
CL(P)	77	46	31	1.65	0.94–2.91
Non-cleft controls	133	63	70		
10-year-olds					
CL(P)	56	25	31	1.89	1.01–3.53
Non-cleft controls	164	49	115		
Total material					
CL(P)	133	71	62	1.89	1.25–2.86
Non-cleft controls	297	112	185		

^a^0-60 % chance to avoid caries

^b^61–100 % chance to avoid caries

**Table 4:** Caries frequency (mean and SD) in relation to risk category. d /D = decayed, m/M = missed surfaces; a primary incisive or canine earlier extracted because of caries was counted as two decayed surfaces and a primary molar was counted as three, f/F filled surfaces, s/S = tooth surfaces. The caries lesions were staged as “initial” (ICDAS 1–2), “moderate” (ICDAS 3–4) and “extensive” (ICDAS 5–6)

Groups	Risk^a^	Low risk^b^
5-year-olds		
*Children with CL(P)*		
dmfs 1–6 (SD)	1.4 (2.9)	0.9 (2.1)
dmfs 3–6 (SD)	1.2 (2.5)	0.8 (2.1)
*Non-cleft controls*		
dmfs 1–6 (SD)	1.2 (3.5)	0.6 (2.5)
dmfs 3–6 (SD)	1.0 (3.0)	0.5 (1.8)
10-year-olds		
*Children with CL(P)*		
dmfs 1–6 + DMFS 1–6 (SD)	1.8 (2.5)	1.2 (1.8)
dmfs 3–6 + DMFS 3–6 (SD)	1.6 (2.3)	1.1 (1.8)
*Non-cleft controls*		
dmfs 1–6 + DMFS 1-6	3.5 (4.5)	1.0 (2.3)
dmfs 3–6 + DMFS 3-6	3.2 (4.1)	0.9 (2.2)

^a^0-60 % chance to avoid caries

^b^61–100 % chance to avoid caries

## Discussion

To the best of our knowledge, this was the first study to apply the Cariogram caries risk assessment model to children with CL(P). In the original manual, five risk categories were advocated but in the clinical-practical context, two risk categories, low risk vs. some risk, are pertinent for the clinical decision-making and patient-centered prevention. This was the reason to merge the original moderate, high and very high categories and compare them with the low and very low risk categories, assuming that preventive “over-treatment” is somewhat more acceptable than neglecting professional preventive care to those with a true need. The modification of the scores entered into the program was made to match the age groups and children living in Sweden and may not be relevant or applicable elsewhere. For example, according to a national survey, 60 % of the 12-year-old children were free from cavities (DMFS = 0) [23]. Therefore, also early enamel lesions were considered in the algorithm “past caries history” in our study which was in contrast to the manual. Furthermore, the stimulated saliva secretion rate was not included in the Cariograms of the youngest age group. Many of the 5-year old children hesitated to cooperate with the collection procedure and obtained secretion rates were most often considered non-reliable. On the other hand, true hyposalivation is relatively uncommon among preschool children [24] so its influence on final Cariogram was likely limited. Moreover, an earlier study using Cariogram in schoolchildren have shown good validity despite omitted saliva secretion rates [25]. The 10-year- old children followed more easily the instructions to chew and spit and the cut off for normal saliva secretion was set to 0.5 ml/min as suggested by Sreebny 2000 [26]. Furthermore, all children living in Sweden with CL(P) and without CL(P) are given all medical and dental treatment free of charge.

The main findings of the present study were that a significantly higher proportion of CL(P) children displayed increased caries risk and the background variables that differed between the groups were oral hygiene and salivary lactobacilli counts. Thus, the null hypothesis was rejected. The fact that the oral hygiene may be jeopardized and impaired in CL(P)-children has been suggested in many previous studies [2, 5, 8]. This can be a result of fear of brushing around the cleft area, the anatomy of the cleft or a loss of elasticity of the surgically repaired lip [2]. Other reasons can be restricted access for tooth brushing and natural cleaning since limited dental arch space attributed to the underdeveloped maxilla and higher incidence of supernumerary teeth cause malalignment of the teeth [12]. There are also earlier reports that the levels of caries-associated bacteria may be elevated in oral cleft children [11, 14]. We were unable to find increased mutans streptococci counts in the CL(P) children but more children with CL(P) displayed high and medium counts of salivary lactobacilli than children in the control groups. The reason for this is not clear but impaired circumoral soft tissue movements in children with clefts [27] can prolong oral clearance time and favor the growth of aciduric bacteria [14]. Therefore, further studies on the oro-facial function in children with clefts would be of interest. Furthermore, untreated and open caries lesions are associated with increased lactobacilli counts [28]. The frequent occurrence of enamel defects (hypoplasia and hypomineralisation) in this CL(P) material [6] can also act as retention sites for plaque and may contribute to the elevated counts of salivary lactobacilli. However, in the Cariogram model, the lactobacilli counts are entered to reflect the “sugar amount” in the diet but, unfortunately, we had no detailed information on the dietary habits in the present study groups.

A considerable number of the children in the present material had chronic diseases that may influence caries risk such as asthma [29], congenital heart disease [30], obesity [31], diabetes [32] and attention deficit hyperactivity disorder and autism [33–35]. Although the proportion of affected children was slightly higher in the CL(P) group (19 %) than in the control group (19 vs. 12 %; NS), this could not fully explain the differences in caries risk between the groups. The findings of the present study reinforce the assumption that children with CL(P) may be regarded as caries risk patients on a group level but an individual risk assessment is needed to tailor and target the need of preventive action. Thus, the Cariogram, or any other structured risk assessment model, should be included in the toolbox of the multi-professional team involved in the comprehensive care of CL(P) children. It should however be underlined that this study did not aim to validate the Cariogram in CL(P) children. For this, a longitudinal design with follow-up examinations is required.

## Conclusion

The present findings demonstrated that 5- and 10-year-old children with CL(P) more often displayed caries risk than age-matched controls. The significant determinants in the Cariogram model were impaired oral hygiene and elevated salivary lactobacilli counts. The results suggest that an objective and structured caries risk assessment model should be applied in the CL(P) care as a basis for the clinical decision-making and individual implementation of caries preventive measures.

## Abbreviations

CL(P): Cleft lip and or palate
QH-Index: Modified Quigley-Hein Index
ICDAS-II: International Caries Detection and Assessment System
QH: Modified Quigley-Hein plaque Index
FORSS: Medical Research Council of Southeast Sweden
Futurum: Academy of Health and Care Jönköping County Council
